# An exploration of acceptability of a collaboratively developed model of formulation

**DOI:** 10.1371/journal.pmen.0000718

**Published:** 2026-09-25

**Authors:** Aneita Pringle, Jonathan Totman, Tine Van Bortel, Emma Kaminskiy

**Affiliations:** 1 School of Psychology and Sport Sciences, Anglia Ruskin University, Cambridge, United Kingdom; 2 University Counselling Service, University of Oxford, Oxford, United Kingdom; 3 Cambridge Public Health Interdisciplinary Research Centre, Department of Psychiatry, University of Cambridge, Cambridge, United Kingdom; 4 Health Innovation East, Cambridge, United Kingdom; James Cook University - Singapore Campus, SINGAPORE

## Abstract

Collaborative, recovery-oriented psychological interventions are in demand within mental health settings, but layers of barriers can impede their implementation, particularly within acute services. Psychological formulation is a recovery-oriented, collaborative process involving the development of a shared understanding of a service user’s mental health difficulties. Psychological formulation has the potential to support a shift away from the embedded medical model; however, few, if any, frameworks have involved service users at the development stage. This study examines the acceptability of a collaboratively developed model of psychological formulation, piloted with staff within a specialist inpatient setting as well as a wider sample of individuals involved in delivering or receiving mental health services. A theory-driven and collaboratively developed psychological formulation model was developed (the ‘Personal Narrative Model’) and acceptability was evaluated with (1) acute inpatient staff working on an adult ward, and (2) a wider sample of professionals and individuals with lived experience. A focus group (*n* = 7), surveys (*n* = 26), and interviews were conducted (*n* = 8) and analysed using reflexive thematic analysis. The formulation model demonstrated promising acceptability although it was not meaningfully implemented within the acute setting. Three overarching themes are presented from the data: 1) the value of collaborative formulation, 2) implementation barriers, and 3) ‘selling’ and embedding change. Although there is an appetite for more contextualised ways of viewing and supporting mental health, underlying practical, cultural, and attitudinal barriers exist. Practical recommendations are shared for future researchers or practitioners seeking to embed psychological interventions within their applied settings.

## Introduction

Psychological formulation involves applying theory to build a collaborative understanding of a client’s difficulties, which then informs the plan of care [1]. It integrates psychological understandings with collaborative work across disciplines [2] and has been positioned as offering a more contextual, psychosocial alternative to current, predominantly biomedical understandings of distress [3]. Psychiatrists, and other mental health professionals recognise formulation as a core competency [4,5], with clinical psychologists viewing this process as a central and essential feature of clinical work [2]. Formulation can take different forms; for example, multidisciplinary team formulation can also be helpful for building understanding and care-planning [6].

Psychological formulation aligns with the recovery model of mental health, which can be described as the accommodation of distress by shifting emphasis from ‘clinical’ outcomes to those personally meaningful to service users [7,8]; it has been endorsed by the UK government for several years. Nevertheless, routine practices such as acute care-planning vary substantially in their alignment with recovery principles [9]. Although service user involvement is a key factor in effective implementation of recovery practices, its integration into practice remains inconsistent [10].

Despite the theoretical value of practices like formulation, barriers introducing alternatives to usual models of care in inpatient settings are well documented [10], including resistance among teams to depart from biomedical orientations [11]. The medical model or ‘biomedical’ model has been found to dominate constructions of mental health in inpatient settings [12], and tends to entail the view that physiological differences can best explain individuals’ mental health difficulties. The medical model tends to foster blaming discourses of service users’ behaviour, reifying the stigmatisation of diagnoses such as borderline personality disorder (BPD, 3) and limiting possibilities for relational growth, whereas psychological formulation has the potential to positively influence interactions between professionals and service users [2].

Aligned with principles of epistemic justice [13] and the disability rights movement’s “nothing about us without us,” psychological formulation emphasises active service-user involvement [2]. Although existing formulation models draw largely from psychological theory and practice, few have been co-designed with service users. Notably, Yeandle and colleagues’ [14] ‘guided formulation’ remains an exception within specific diagnostic contexts (e.g., borderline personality disorder, BPD). Consequently, service-user involvement at the developmental stage remains a key gap. Embedding co-production from the outset enables service users to shape the framing, assumptions, and process of formulation and supports recovery-oriented values such as autonomy and collaboration.

The Power Threat Meaning Framework [15] has contributed significantly to emphasising meaning-making and contextual understandings of distress. Developed collaboratively with service users, clinicians, theory, and research, the PTMF offers a conceptual alternative to diagnostic models by shifting the question from “What is wrong with you?” to “What has happened to you?” As a meta-theory, the PTMF invites adaptation into local practice. Given the barriers to implementing alternatives to the biomedical model in inpatient settings, adapting the PTMF collaboratively with service users and staff may support the development of a formulation model grounded in lived realities and sensitive to contextual influences on mental health.

To address this gap, the present study reports on the co-development and acceptability of the Personal Narrative Model (PNM), a new formulation model developed with clinicians, academics, and a Service User Advisory Group (SUAG). The PNM represents one of the few known formulation frameworks involving service users during initial development. This paper examines the acceptability of the PNM, alongside enablers and barriers to its implementation, from the perspectives of specialist inpatient staff, mental health practitioners, and individuals with lived experience.

## Materials and methods

### Ethics statement

Ethical approval for this study was obtained from the North West Liverpool Central Research Ethics Committee (formerly Liverpool East Research Ethics Committee; REC reference 18/NW/0212; IRAS ID 227356) and the Anglia Ruskin University Faculty Research Ethics Panel (reference EHPGR-28). All participants provided written informed consent prior to participation in the study.

### Study design

**Fig 1:**
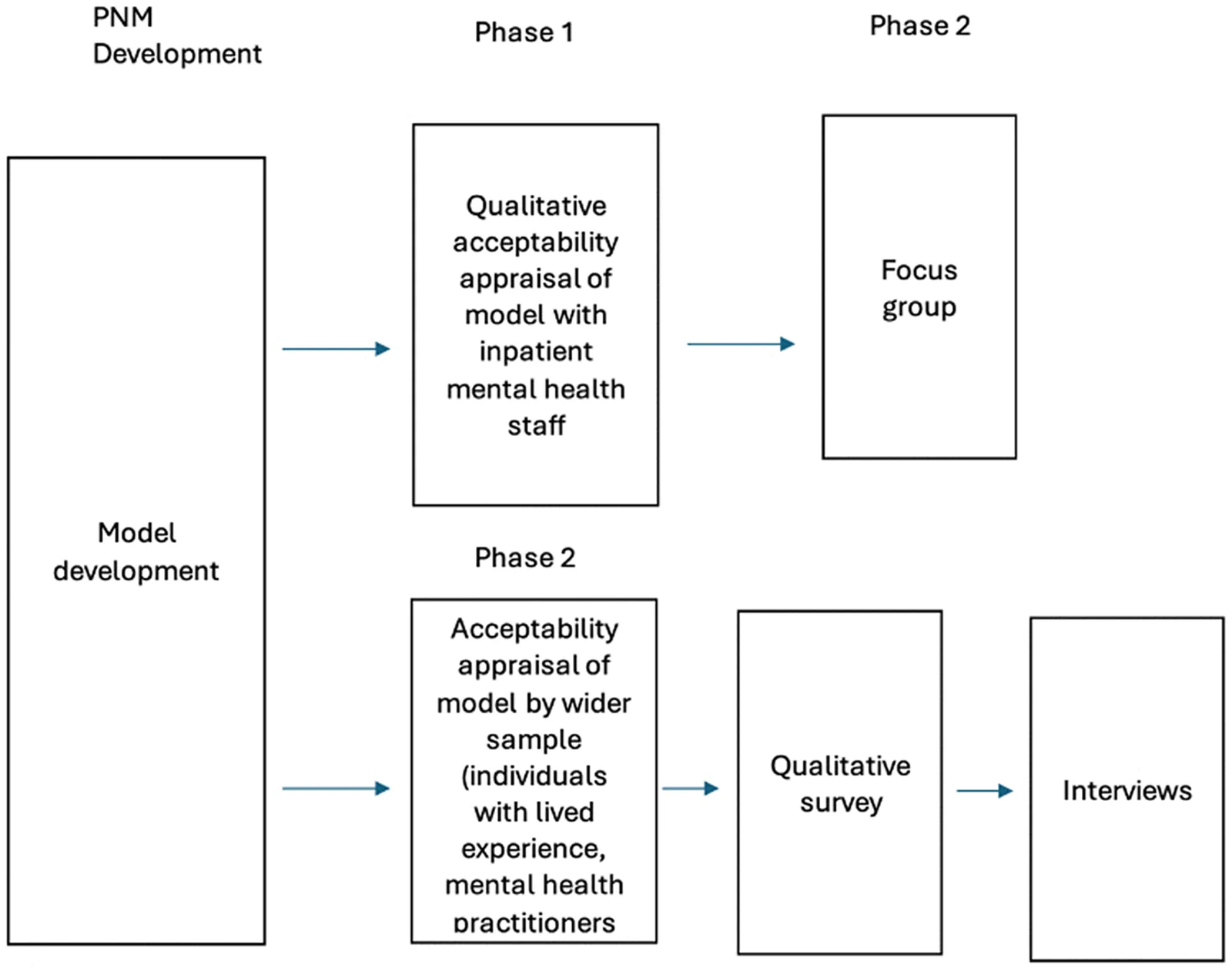
The process of data collection.

This study evaluated the acceptability of the PNM using a two-phase exploratory sequential design, where the results of Phase 1, including the barriers reported, were fed back to and explored with participants in Phase 2. Questions included how the results found with ward staff related to their own experiences of services (see Fig 1). This is reported in accordance with COREQ [16]; please see supplementary information S1 Checklist for the COREQ checklist. A critical realist framework guided the work, positing that an external reality exists independently of our perceptions [17] and can be approached using methodological pluralism. The study formed part of AP’s doctoral research. A Service User Advisory Group (SUAG) supported all stages of the research design, including the development of participant materials and the interview schedule.

### Theoretical underpinning of the PNM

The PNM (S1 Fig) was developed to offer an accessible and contextualised approach to understanding distress with the inpatient setting in mind. It aimed to avoid the limitations of a rigid, exclusively theory-driven model developed in the absence of coproduction by prioritising meaningful engagement and co-production with service users, practitioners, and academics. The model is designed for use either one-to-one with service users or within team formulations. It is transtheoretical, strengths-focused, and uses accessible language while integrating political, social, and biopsychosocial influences on distress. The model aims to promote epistemic justice [13].

The PNM was co-designed for use on an acute inpatient unit by service users and mental health practitioners. It incorporates the PTMF (Johnstone et al., 2018) and Hagan and Smail’s [18] power-mapping model, as well as strengths-based approaches [19,20], the CASIG framework [21], the Comprehend, Cope and Connect model [22,23], and established team formulation guidelines [24–26]. The PNM offers prompts for reflection rather than a fixed protocol, with headings such as ‘Strengths and Resources,’ ‘Past Experiences,’ ‘Meaning,’ ‘Threat,’ ‘Threat Responses,’ and ‘Next Steps.’

### Co-design procedures and contributions

Two core SUAG members attended approximately 20 meetings during model development and contributed to the wider research programme. Additional individuals with lived experience contributed on an ad hoc basis, alongside academic and clinician collaborators. Three iterative versions of the PNM were developed. Academics encouraged a multimodal, person-centred design; the SUAG emphasised grounding the model in ward realities, strengthening its containing qualities, prioritising accessibility of language, and promoting strengths-based practice.

### Study setting, recruitment, and participants

The PNM was piloted on a UK-based specialist inpatient ward (approximately 10 beds) for individuals diagnosed with a personality disorder. The multidisciplinary team included ward managers, clinical nurse specialists, an occupational therapist, nurses, and support staff. AP introduced the research as part of a doctoral project, its purpose and, delivered four 1.5‑hour training sessions. A clinical psychologist member of the research team (JT) co-delivered a session focused on ‘Ways Forward,’ specifically exploring and discussing how the PNM might practically be implemented within routine practice and facilitated a role-play exercise simulating how the PNM might be discussed with a service user. Training covered the development and use of the PNM, the research process, formulation practice, and identification of enablers and barriers to implementation. Phase 1 recruitment took place April 2019 until November 2019, while Phase 2 recruitment too place May 2020 until November 2020.

Staff were then invited to trial the PNM in practice, developing formulations alongside service users in one-to-one sessions or as a team.

Acceptability data were collected in two phases.

**Phase 1:** a focus group with seven inpatient staff (three nurses, one clinical nurse specialist, one healthcare assistant, one occupational therapist, one declined to complete the demographics form; average length of training = 6 years).

**Phase 2:** an online survey (the qualitative results alone are explored here; see supplementary information S1 File for the full item set) with a broader UK sample (*n* = 26). This included seven mental health practitioners, five mental health practitioners with lived experience (average length of training = 5 years) and follow‑up interviews (*n* = 8; four with lived experience, four practitioners including one with lived experience). Fourteen individuals with lived experience also participated in the survey; eight preferred not to say the type of support or services they had received while of the remaining six participants, one attended perinatal services, one “adult mental health services,” one “NHS Mental Health Assessments and Therapy Services” and one “CAMHS” (Child and Adolescent Mental Health Services, now Children and Young People’s Mental Health Services). Two participants had attended multiple services (one having attended “CAMHS, [NHS Trust], IESO [now known as Mindler, UK]” and one “Child and adolescent mental health services, adult community mental health services, improving access to psychology therapy, MIND counselling”. No participants withdrew.

Phase 1 recruitment was facilitated by ward managers while Phase 2 recruitment occurred via social media, professional networks, and the university’s SONA system. A convenience sampling approach was used. Although conducted in two phases, all data were combined during analysis. The reason for combining the datasets was the exploratory sequential design, meaning the results of Phase 1 fed into those of Phase 2, and are therefore dependent and linked. There was also substantial overlap between the two datasets.

Ethical approval was obtained for both phases (18 NW 0212, IRAS ID 227356; EHPGR‑28). All participants provided written informed consent.

### Procedure

Phase 1 data collection occurred one month after staff training and involved a 50‑minute audio-recorded semi-structured focus group facilitated by AP, a trained female doctoral student. The focus group took place in a room on the ward.

In Phase 2, the design and implementation was shifted online as an adaptation necessitated by the COVID-19 pandemic. Participants viewed video introductions to the PNM and the research (see supplementary information S1 Video) and completed open-ended survey questions (e.g., “How acceptable would you find the PNM for distress?” and “How likely is the PNM to make improvements?”). Participants could also opt into a semi‑structured interview (*n* = 8), conducted via a recorded video call, facilitated by AP. Interviews lasted between seven minutes (technical fault; retained in analysis, but given a proportional amount of weight given only one question from the interview schedule was answered) and 61 minutes. Notes were taken throughout data collection.

### Data analysis

AP transcribed and analysed all data inductively using reflexive thematic analysis following Braun and Clarke’s six stages [27,28]. Two research team members double‑coded selected transcripts. NVivo 12 supported data organisation. Consistent with a reflexive approach, data saturation was not sought [27]. Pseudonyms were assigned to all participants. One duplicate survey participant was identified, and their responses were combined.

Reflexivity and trustworthiness were supported by transparency regarding AP’s position as a doctoral researcher. The member reflection process with Phase 1 staff involved a presentation of the themes at the point of reviewing them, delivered remotely with ward staff as a group. Staff verbally provided positive feedback on themes; feedback concerned the number of themes at the time which were then reduced through the analysis process. Had divergent feedback been received, AP would have taken this back to the research team and asked to re-present the amended themes to ward staff. AP took on multiple roles throughout this research and her investment in the PNM is likely to have influenced decision-making at key stages of the design and analysis. This was mitigated through the maintenance of a reflexive journal and supervision.

## Results

### Survey results

Participants expressed mixed views regarding whether the PNM represented a shift from their usual ways of thinking about mental health. In response to the question, “Is the PNM different to how you currently think about mental health?”, 12 participants responded “Not at all” (including two practitioners and four practitioners with lived experience), 11 responded “Somewhat,” one “Reasonably,” and three “Mostly.”

### Themes

Three themes captured participants’ views regarding the acceptability of the PNM and the barriers and enablers influencing its implementation. These included:

1. The value of collaborative formulation
2. ‘Selling’ and practical ways to embed change
3. Implementation barriers, including the sub-themes challenging attitudes and practical concerns.

#### Value of collaborative formulation.

Participants across groups responded positively to the PNM (‘incredibly useful and needed,’ Survey, Jane, lived experience), (‘I’d be so happy to use it’ Interview, Jessica, practitioner). They valued the grounding of the model in everyday inpatient experiences, an element specifically emphasised by the SUAG.

It also looks at how they are feeling about current treatment which is something that is not often talked about in therapy sessions.

(Interview, Yasmine, lived experience)

Clinician participants also appreciated that the model supports exploration of individual meaning-making.

I think it gives you more of an opportunity to think about the person’s constructs which some other models don’t really encourage as much.

(Interview, Delia, practitioner)

Some participants described the PNM as helpful for clinicians and service users as a way to establish a framework to better understand experiences.

If you’ve seen the progression and it literally lets you break your thoughts down into those sections, so you can structure your thoughts so going forward, it’s almost like a little life tool. (Interview, Chloe, lived experience)

Participants indicated the Model could be helpful with care-planning: development of meaning and narrative could prompt reflection about service users’ stories and thereby help identify ways forward. Participants appreciated the model’s whole-person approach and contextualisation of current ways of thinking in relation to past experiences.

The main strength is that it is based on the individual person as a whole, and from their own personal narrative instead of being based on a medical model which is based on numbers on a paper resulting from laboratory tests, exam results, etc. (Survey, Fatima, practitioner).

From experience, people generally benefit from making sense of their current problems with past problems. Not everyone is interested in knowing how the past shaped them; they sometimes just want to focus on what the problem is and how can they fix it. For the other group of people, knowing how the past shaped them and making links with present problems helps them to get to overcome their difficulties. (Survey, Jessica, practitioner)

Both practitioners and individuals with lived experience highlighted the value of a strengths-based approach, finding the balance of negative meaning-making with more positive interpretations especially useful. The quotes below illustrate the PNM was felt to offer benefit both by offering an alternative to the deficits-based medical model, as well as an appreciation of the multiplicity of service user experiences and interpretations.

Focusing on strength aligns with recovery model, giving service users a sense of self, removing labels of illness and top-down care (paternalistic).” (Survey, Chloe, lived experience)

Usually the focus is on the negative outcome of people’s past experience, but with this formulation, the focus is on positive meaning, as if I did not experience this I would not be the person I am now, e.g., bullying, negative: “I am a failure” but positive: “it made me stronger to ‘fight bullies and protect others from bullies.’” […] I am actually going to introduce this to my client as it’s true; it is both meanings. (Survey, Jessica, practitioner).

Some participants felt that focusing on strengths may be challenging or feel invalidating for individuals who struggle to identify positive attributes about themselves.

For some personality disorder clients, the suggestion of any strength is invalidating […]so they tend to resist. It’s like, how dare you say that I’ve got strengths.(Interview, Richard, practitioner and lived experience)

Engaging service users to look for strengths, etc. could be a very hard, possibly distressing and lengthy process due to long term self-worth/beliefs but is essential for long term recovery. (Survey, Chloe, lived experience)

#### ‘Selling’ and embedding change.

This theme describes recommended strategies for promoting the use of the PNM, including its potential benefits, positioning the PNM to address perceived gaps in practice, and adopting specific strategies to overcome resistance. It also describes methods to effectively embed change.

Several ward participants found value in taking part in the process of training on the PNM and being invited to implement it within their practice. Some viewed formulation as ‘too much too soon’ and required additional preparation.

Participants shared strategies to effectively motivate adoption of new ways of working; for example, emphasising the utility of the new model as building on and supporting current practices such as improving confidence to work with additional risk levels. The importance of accessibility, including junior members of staff, was emphasised.

People have been quite apprehensive about doing it [formulation]. I think there just isn’t experience of doing a formulation and viewing a formulation. It’s a very psychological term, whereas actually, I think everyone has the skills to do their formulation. (Focus group, Sydney, practitioner)

One member of staff was considering introducing the model with new service users, and that the PNM remained in their mind following the training.

Ward participants highlighted opportunities for the PNM to enhance or replace existing practices. For example, care-planning was felt to be divorced from otherwise meaningful ways of working.

Sydney: When another organization asks for a copy of a care plan, and there’s like, this is not doing us any justice, this copy […] I hate sending-I hate forwarding it to a community, social work whatever, I find it embarrassing […]

Brooke: It’s literally just a tick box of just writing. (Focus group, practitioners)

Some practitioners felt the PNM offered an opportunity to add meaning and utility to their practice.

Sydney: It can’t just be another document that we do and it just sits there. It kind of has to-have-we would have to think about where its value is helping us as a ward and-

Brandon: I mean, possibly formulation could replace care-planning can’t it? Because one of the aims of it is more formulation of care and processes. (Focus group, practitioners)

#### Implementation barriers.

This theme describes the attitudinal, practical, and risk-management barriers raised by participants, in relation to change more broadly and the PNM particularly.

*Challenging attitudes*. Practitioner participants emphasised the difficulty of introducing change within inpatient settings. For example, Richard (interview; practitioner and lived experience) stated, “I thought you were very brave doing it on an inpatient, to be honest” due to the inpatient wards’ reputation as busy, high-risk, and inhospitable; Richard suggested this is particularly so in recent years due to “a lot less informal admissions,” implying attendant rigidity in restrictive ways of working and barriers to collaboration given a culture of risk aversion.

Participants acknowledged the medical model is more deeply embedded within inpatient practices and that this presents a specific challenge for implementation of the PNM. For example, in Lindsay’s (practitioner) survey response, she indicated:

It can be very tricky to implement anything new on an inpatient setting as it is still (unfortunately) medicalised. Staff are busy putting out fires, they don’t have time to look for the source. (Survey, Lindsay, practitioner).

In her interview, she contrasted community teams with inpatient settings:

In a community team, I think it’s much easier than on an inpatient ward. Whereas a lot of the staff I’ve come across it’s more, ‘well, they just need – we just need to check with the meds and they’ll be fine.’

Practitioner participants felt inpatient staff can be resistant to change given the added commitment required: “people don’t want to be there,” (Interview, Richard, practitioner and lived experience) and the absence of incentive of new ways of working. In her survey, Jessica (practitioner) stated “[staff] get stuck in their ways,” on which she then expanded:

I came across lots of nurses who just do it as they can’t get a job elsewhere and as there is a constant demand on employing mental health staff [so] they know that they will always have the job so they don’t want to change their attitude, no matter what is given to them. (Survey, Jessica, practitioner).

Both practitioners and individuals with lived experience reported that pushing for unpopular change is inadvisable.

If you try to bring on change and people don’t like it because it will give them more work to do, people will get bullied and people lose jobs… if you want to keep your job sometimes you need to be qui-you need to shut up, basically.

(Interview, Jessica, practitioner)

“I’ve heard things on the – on [social media] you know, it can be daunting, intimidated; you could lose your job if you take these kind of things upon yourself.”

(Interview, Chloe, lived experience)

Some participants raised potential barriers to collaborative formulation, including scenarios where service users do not wish to actively engage. For example, Jane explained, “If I was feeling wilful, then I wouldn’t use it, if you know what I mean? Like, if I was feeling like I want somebody else to fix this for me.” (Interview, Jane, lived experience).

Richard agreed with this concern:

Sometimes you come across clients that basically say I don’t wanna get involved in this; just cure me. Just problem solve my stuff for me. (Interview, Richard, practitioner and lived experience)

*Practical concerns*. Staff expressed concern about additional burden, although it was suggested service users take on the work themselves. One member of staff raised nurses might not feel formulation fits within the remit of their role, suggesting it is the responsibility of those trained in psychological approaches.

So I can imagine for nurses it would be just ‘another thing to do’ that they generally feel psychologists should do. (Interview, Jessica, practitioner)

Some participants felt there were drawbacks to the implementation of the PNM, including limited staff availability and the need to train individuals on the use of PNM requiring further resourcing within already stretched settings. Concerns were noted about the introduction of conversations around, for example, staff availability, which cannot be altered:

There’s a lot of things that we can’t change, like staff availability, the regular care-planning reviews. A lot of this stuff kind of almost happens within the environment. And so for me, I would be quite nervous to raise that. (Interview, Richard, both practitioner and lived experience).

Richard further described a solution- or goal-focused orientation within NHS services that chafes at the discussion of problems mental health staff cannot immediately help manage, such as staff availability or physical health challenges, speculating this would impede uptake of PNM.

There was also concern among participants, particularly staff, in interviews and the focus group that speaking of past experiences might introduce additional risk staff feel unequipped to manage. A few participants were also concerned about destabilising the services user, advising to ensure support is available and consider the timing.

It depends on what their presenting difficulties are. If they’re presenting with high risk … I wouldn’t encourage them to talk so much about their past experiences for the fear of further destabilising them. (Interview, Delia, practitioner)

I would probably check beforehand, like, element of risk, I suppose. Obviously that depends on the timing of the person and where they are. At certain points in my mental health world, there’s no way I could’ve talked about my history without it causing some significant distress. (Interview, Jane, lived experience)

Overall, the concerns raised by participants relating to risk were dependent on training or support and timing, ensuring adequate consideration is given to both to safely introduce PNM.

## Discussion

The PNM, one of the few known formulation models to incorporate service users in its development phase, was acceptable to participants and provided practical utility in helping them structure and understand service users’ experiences. It also supported meaning-making around service users’ experiences and enhanced the perceived coherence of the care model. Overall, the PNM was viewed favourably by participants.

Although participants valued the collaborative formulation approach, interestingly at the time of the focus group no participants indicated they had adopted the PNM in their practice, barring one participant who indicated they intended to practice the PNM. This may reflect an important implementation gap; several such implementation barriers were identified, including concerns about discussing past experiences and staff resistance to adopting new practices. Inpatient staff described both practical and attitudinal challenges, many of which were specific to the inpatient context. Participants highlighted the importance of strategies to promote change, such as supporting staff based on their existing level of knowledge and familiarity with psychological interventions.

A key finding concerned the perceived value of formulation as a potential alternative to care-planning, which was commonly described as lacking meaning and failing to represent the quality of work undertaken on the ward. This is notable given broader concerns about the purpose and usefulness of current care-planning processes [29] and the growing interest in alternative approaches such as formulation. However, in the context of evolving and resource-limited inpatient mental health services, formulation would need to be integrated into usual care—or replace current practices—to avoid being perceived as an additional task. Resistance to new ways of working within inpatient environments is well recognised [30]. Team formulations may be positioned to offer additional benefits for staff teams; for example, they have been shown to enhance psychological understanding of service users and improve consistency in staff perspectives [31]. Lake [30] also suggested that psychologists may support multidisciplinary teams to develop their own formulations, thereby broadening access to formulation skills.

Barriers to new ways of working have been identified in previous research on implementation within inpatient settings, such as busy ward environments, training needs, and high levels of distress [32]. More recent evidence indicates that these barriers may be heightened when proposed changes diverge from a predominantly biomedical model [10]. Consistent with findings from this study, [32] systematic review highlighted leadership support and prioritisation of therapeutic relationships as key facilitators of successful implementation.

Staff concerns about risks associated with discussing potentially distressing past events—particularly fears about being unable to manage service user distress—may help explain why many individuals report not being asked about their histories while accessing services. A systematic review of international literature by Read and colleagues exploring enquiry by adult mental health services of childhood adversity [33] found that only 0–22% of service users reported being asked about historical trauma, despite widespread recognition that many individuals have experienced abuse or trauma [34].

### Strengths and limitations

The PNM is among the few formulation models developed collaboratively with individuals who have lived experience. The SUAG was actively involved throughout the process. The PNM may offer a recovery-oriented framework to support more collaborative care-planning.

However, several limitations should be noted. Qualitative data were analysed by a single researcher, and the sample sizes were small [35] and contextually limited. Only one site, an adult inpatient setting, was available for the study, and recruitment challenges were encountered in both the pilot intervention and the interviews. While such challenges are common in mental health services research, greater site diversity (e.g., geography, ward type, population characteristics) and a larger survey/interview sample would likely have strengthened the rigour and depth of the findings regarding acceptability across different settings. Survey wording relating to perceptions of the PNM may have leant towards a positive frame, e.g., “How likely is the PNM to make improvements?” Such demand characteristics may have implicitly communicated to participants how they were ‘expected’ to respond and thus also framed subsequent responses.

## Conclusion

The PNM, among the first known formulation models to involve service users in the development stage, was acceptable to participants including ward staff, people with lived experience, and mental health practitioners. Both practical enablers and barriers were identified relating to the shift away from the medicalised model, offering valuable insights and recommendations for integrating formulation or other recovery-focused interventions within acute inpatient environments and beyond.

## Supporting information

S1 Checklist COREQ checklist. (PDF)

S1 Fig PNM Model. (TIF)

S1 File Online survey items. (PDF)

S1 Video Video explanation of the PNM and of Phase 1 results. (PDF)
