# Supplementary material for: An exploration of acceptability of a collaboratively developed model of formulation
Source: PLOS Ment Health. 2026 Sep 25;3(9):e0000718. doi: 10.1371/journal.pmen.0000718 (PMC13614565; doi:10.1371/journal.pmen.0000718)
Supplement: Supplementary file 2 [file pmen.0000718.s003.pdf]

PNM Acceptability Survey

1. **Age**

18-25	25-49	50-64	65+	Prefer not to say
1	2	3	4	5

2. **Gender identity**

Male	Female	Non-binary/third gender	Prefer to self-describe	Prefer not to say
1	2	3	4	5

3. **How would you describe yourself?**

***Note: those who select 'both' will be directed to respond to questions related to mental health practitioners**

I am a mental health practitioner	I have lived experience of mental health difficulties	I am both a mental health practitioner AND I have lived experience of mental health difficulties
1	2	3

IF Mental health practitioner

4. **How many years of training have you undergone?**

Please indicate (Free text)	Prefer not to say

5. **How many years have you been in your current role?**

Please indicate (Free text)	Prefer not to say

6. **How would you describe your employing organisation?**

Private practice	NHS	Counselling agency	Third sector	Other	Prefer not to say
1	2	3	4	5	6

7. **What type of setting do you work in?**

Please indicate (Free text)	Prefer not to say

8. **In what part of England do you practice?**

South East	South West	London	East of England	East Midlands	West Midlands	Yorkshire and the Humber	North West	North East	Wales	Scotl
1	2	3	4	5	6	7	8	9	10	11

IF individual with experience of mental health

9. **In what part(s) of England have you received services?**

South East	South West	London	East of England	East Midlands	West Midlands	Yorkshire and the Humber	North West	North East	Wales	Scotl
1	2	3	4	5	6	7	8	9	10	11

10. What type of support or services have you received (e.g. child and adolescent mental health services, etc.)?

Please indicate (Free text)	Prefer not to say

11. Please indicate the duration of your experience of mental health difficulties

Please indicate (Free text)	Prefer not to say

12. Please indicate whether you are currently undergoing treatment from mental health services.

Yes	No	Prefer not to say

Training Feedback Schedule
Questions about the PNM
ALL participants

1. **What are your thoughts about the Model? (For example, what are the stronger and/or weaker parts?) (Free text)**
2. **How acceptable would you find the Personal Narrative Model to help treat mental health distress?**

Very acceptable	Acceptable	Slightly acceptable	Moderately acceptable	Slightly unacceptable	Unacceptable	Not at all acceptable
1	2	3	4	5	6	7

3. **How consistent is the Personal Narrative Model with your common sense or everyday notions about a care planning tool for mental health should be?**

Very consistent	Consistent	Slightly consistent	Moderately consistent	Slightly inconsistent	Inconsistent	Very different or inconsistent
1	2	3	4	5	6	7

4. **To what extent do you think there might be risks in undergoing the Personal Narrative Model?**

Lots of risks are likely	Risks are likely	Risks are slightly likely	Some risks are likely	Risks are slightly unlikely	Risks are not very likely	Nor isks are likely
1	2	3	4	5	6	7

5. How much do you like the steps or factors involved with the Personal Narrative Model?

Do not like them at all	Somewhat dislike them	Slightly dislike them	Moderately like them	Slightly like them	Somewhat like them	Like them very much
1	2	3	4	5	6	7

6. How effective do you think the Personal Narrative Model is likely to be?

Not at all effective	Somewhat ineffective	Slightly ineffective	Moderately effective	Slightly effective	Somewhat effective	Very effective
1	2	3	4	5	6	7

7. How likely is the Personal Narrative Model to make permanent improvements to mental health?

Unlikely	Somewhat unlikely	Slightly unlikely	Moderately likely	Slightly unlikely	Somewhat likely	Very likely
1	2	3	4	5	6	7

8. To what extent do you think the downsides are likely to result from the Personal Narrative Model treatment?

Many downsides likely	Downsides likely	Several downsides likely	Some downsides would occur	A few downsides likely	A couple downsides likely	No downsides likely
1	2	3	4	5	6	7

9. How much discomfort do you think is likely to be experienced during the the development of the Personal Narrative Model?

Very much discomfort	Mostly discomfort	Much discomfort	Moderate discomfort	Some discomfort	Slight discomfort	No discomfort at all
1	2	3	4	5	6	7

10. Overall, what is your general reaction to the Personal Narrative Model?

Very negative	Negative	Slightly negative	Ambivalent	Slightly positive	Positive	Very positive
1	2	3	4	5	6	7

11. How could your understanding of the Personal Narrative Model be improved, if at all? (For example, further training, practice using the Model)? (Free text)

12. Is the Personal Narrative Model different from how you currently practice/think about mental health?

Not at all	Somewhat	Reasonable	Mostly	Very
1	2	3	4	5

13. Can you say more about how it’s similar or different? (Free text)

IF mental health practitioner

14. How relevant do you think the Personal Narrative Model would be in your service?

Not at all	Somewhat	Reasonable	Mostly	Very
1	2	3	4	5

15. How confident would you be to use the Personal Narrative Model?

Not at all	Somewhat	Reasonable	Mostly	Very
1	2	3	4	5

16. What would help you feel more confident to use the Personal Narrative Model? (Free text)
17. Do you have any other thoughts you would like to share? (Free text)

Phase 2

***Note to clarify: IF mental health practitioner, they will skip the below questions, resuming at Video 2 and related questions**

IF individual with experience of mental health difficulties

1. Thinking about the last time you received services, were you encouraged to tell your story?

Yes	No	Prefer not to say
1	2	

2. Have you worked together with staff to understand your difficulties before?

Yes	No	Prefer not to say
1	2	

3. Have you worked together with staff to understand your strengths before?

Yes	No	Prefer not to say
1	2	

4. Has personal meaning been discussed with you in care-planning?

Yes	No	Prefer not to say
1	2	

5. How involved have you been in your care-planning?

Not at all	Somewhat	Reasonable	Mostly	Very
1	2	3	4	5

6. How could you have been supported to be more involved in your treatment? (Free text)
7. Would you like to say more about your experience of treatment and how it could have been improved? (Free text)

***Note to clarify: IF individual with experience of mental health difficulties, they will skip Video 2 and related questions**

IF practitioners

Questions about the training/explanation

1. What were your thoughts about the research results? (Free text)
2. How do you think I could have improved recruitment? Attendance? Outcome measure collection? (Free text)
3. Did the themes from the focus group align with your experience? How so? (Free text)
4. What questions came up for you as you were watching the videos? (Free text)

Interview/Focus Group schedule

*These may change/be supplemented based on the nature of responses on to the Model video and/or how the interview/focus group proceeds.

Before the interview/focus group

- Thanks so much for taking the time today and also for your participation in the first part of the study!
- Where are you based?
- How did you hear about the study?
- Explain what the interview/focus group is about, referring to information sheet, what the purpose of the interview is
    - So the purpose of this research is to enhance the Personal Narrative Model, which was developed in previous iterations of research; I'm hoping to get a broader sense of acceptability and suggestions for improvement from outside the original research setting
    - Everything you say will be kept confidential but for the usual exceptions regarding safety and risk, which I don't anticipate being likely here
    - I'd like to talk to you to generate suggestions for enhancing the programme and implementation of the Model
- How long do you have?
- Explain there are no right or wrong answers
- Reiterate people can take breaks if/when needed and how to alert me
- If lose connection, my first port of call will be to swap to data and if that fails, is it okay to conduct over phone? Just in case, my mobile number is XXX.
- Remind participants about confidentiality
    - With your permission, I will be recording interviews and focus groups using Teams' recording function. I will transcribe the discussion (with identifying details altered or removed), then delete the recording. All data will be password-protected and stored securely in the European Economic Area.
- Answer any questions
- Begin recording on video-chat platform

* What were your initial thoughts about the PNM?
* Imagine you'd like to introduce the PNM in your context. What factors do you think would influence the implementation?
* What would be the main barriers to applying PNM in your context?
* What would be the main enablers of the implementation of the PNM in your context?
* What has been helpful in implementing new practices in your work/practice?
    * (Life for participants with lived experience of mental health difficulties, practice for practitioners)
* How could service users be involved in the implementation?
* What is the likely course of future implementation?
    * What do you think the timeline would be; what do you think initial reactions would be; etc.
    * What would be other staff reactions? Service user reactions?
    * Examples of how an implementation went for you in your current/previous role(s)?
* How does the PNM contrast with current practices in your context?

- Can you see any ways of preventing it being ‘co-opted’ within the existing system?
- What cultural needs do you think are overlooked by standard interventions that should be included?
- What would it take for the Model to be more culturally inclusive?
- Is there anything else you would like to share or discuss?

How could support to make more understandable?
Narrative can be quite emotional, distressing, painful. What sorts of things could support people to work through?
