# Supplementary material for: An exploration of acceptability of a collaboratively developed model of formulation
Source: PLOS Ment Health. 2026 Sep 25;3(9):e0000718. doi: 10.1371/journal.pmen.0000718 (PMC13614565; doi:10.1371/journal.pmen.0000718)
Supplement: Supplementary file 3 [file pmen.0000718.s004.pdf]

Supporting information

S1 Video: Video explanation of the PNM and of Phase 1 results

Video 1 link

https://youtu.be/0bvqd7VyIkw

Video 2 link

https://youtu.be/U5aXRtmsPmY
